# Model-Based Analysis and Regulating Approach of Air-Coupled Transducers with Spurious Resonance

**DOI:** 10.3390/s20216184

**Published:** 2020-10-30

**Authors:** Xiangxiang Peng, Liang Hu, Weiting Liu, Xin Fu

**Affiliations:** State Key Laboratory of Fluid Power and Mechatronic Systems, Zhejiang University, Hangzhou 310027, China; pengxiangx@zju.edu.cn (X.P.); cmeehuli@zju.edu.cn (L.H.); xfu@zju.edu.cn (X.F.)

**Keywords:** air-coupled transducers, electrical impedance, spurious resonance, equivalent model, pre-tightening force, vibration velocity

## Abstract

As an essential characteristic of air-coupled transducers, electrical impedance can provide valuable information for quality control during manufacturing of transducers. It is also found feasible to directly read the optimal operating frequency from the impedance plots when the resonance is independent of the others. However, the spurious resonance emerges when two neighboring resonances are closely spaced, resulting in distorted impedance and ambiguous optimal operating frequency. In this paper, the electrical impedance of air-coupled transducers with spurious resonance is modeled using the Butterworth–Van Dyke (BVD) equivalent circuit. Then model-based sensitivity analysis is performed to evaluate the mutual interference between adjacent resonances. Based on the analysis results, the prestress method is proposed to regulate and suppress the spurious resonance by adjusting the equivalent parameters of the BVD model. Experimental study was carried out on the response of the electrical impedance and the vibration velocity of the transducer with spurious resonance to pre-tightening force. The results show that the spurious resonance disappeared when the pre-tightening force was initially loaded. Moreover, the vibration velocity of two main resonance peaks increases about 45.6% and 33.9% as the pre-tightening torque increases to 0.25 N∙m. Hence it is validated that the proposed prestress method is efficient to suppress the spurious resonance and improve the transducers performance.

## 1. Introduction

Over the last decades, different kinds of air-coupled ultrasonic transducers have been developed and widely used in ultrasound imaging, non-destructive evaluation, range finding, and flow measurement [1,2,3,4,5,6,7]. Among various air-coupled transducers, the transducers constituted of piezoceramic disc and acoustic impedance matching layers have received extensive attention due to their reliable performance and low environmental dependence [8]. Nevertheless, air-coupled transducers still suffer from low radiation efficiency as a result of nonideal impedance matching between piezoceramic and air medium. Compared to water-borne transducers, air-coupled transducers possess higher quality factor (*Q*) and narrower bandwidth [9]. Consequently, the operating frequency of air-coupled transducers requires careful arrangement especially when working in pairs [10]. In general, a transducer has the maximum transmitting efficiency at its series resonance frequency and the maximum receiving efficiency at its parallel resonance frequency [11]. It is found feasible to directly read the optimal operating frequency from the impedance or admittance plots when there is no strong coupling between adjacent resonances. However, the impedance of actual transducers somehow exhibits two or more resonant modes at the frequency where a single main resonance is expected [12,13,14,15]. The undesired resonance in the vicinity of a main resonance is called spurious or parasitic resonance. The occurrence of spurious resonance brings trouble to the determination of the optimal operating frequency [16], thus hindering the subsequent design of excitation scheme, which is detrimental to the optimal use of transducers. Besides, the existence of spurious resonance will lead to poor product performance and consistency.

Regarding the mechanism of spurious resonances, several studies have been carried out and three main influential factors were pointed out. Firstly, the radiation medium has an influence on the impedance of transducers. Spurious resonances are more common in air-loaded transducers than water-borne ones [17]. Moreover, nonideal constituent materials are prone to cause spurious resonances in transducers. Redwood attributed the formation of two coupled modes to a perturbation of symmetry (such as a flaw in the material or imperfect geometry) through experimental study on a rectangular piezoceramic plate [12]. It is suggested that spurious resonance can also be introduced in 1–3 piezoelectric composite transducers due to relatively low loss in high glass transition temperature polymer [18]. Research indicated that inter-pillar modes in 1–3 connectivity piezoelectric composites were generated by Lamb wave propagating through the substrate between pillars [19]. These spurious modes become prominent when their frequencies approach those of the fundamental thickness mode and its harmonics. Last but not least, the existence of the test fixture clamping force will also result in spurious resonance in a piezoceramic plate [20]. Even with these studies, it is still not possible to fully understand the generation mechanism of spurious resonance in air-coupled transducers. The core of an air-coupled transducer is mainly composed of a piezoelectric ceramic disc and an acoustic matching layer, and is bonded with glue. Then a whole transducer is made after the core being encapsulated into the housing. As a multicomponent adhesive assembly, things become more intricate on the spurious resonance problem.

To address the spurious resonance problem, further studies have been triggered. Through measurement and FEM/BEM (finite element method/boundary element method) simulation of transverse effects in SAW (surface acoustic wave) resonators, Solal et al. confirmed that small gaps and dummy electrodes contributed to the reduction of spurious responses [21]. Hammarstrom et al. put forward a suppression approach of spurious resonances by using a kerfed transducer instead of a solid one and hence facilitating the determination of the optimal working frequency [16]. To eliminate spurious oscillations such as Lamb waves and bulk shear waves generated by normal components of electric field, Zaitsev et al. have suggested to separate the source of the HF (high frequency) electric field and resounded piezoelectric plate by air gap in lateral electric field excited piezoelectric resonator [22]. Zou et al. designed a transverse spurious resonance free AlN Lamb wave device employing piston mode structure by adding a slow border region (“hammerhead”) at the edge of the active aperture [23]. The spurious resonance free devices with high *Q* and large effective electromechanical coupling factor ($k_{eff}^{2}$) reduce the additional transversal loss and show no performance degradations of the main mode. These studies mainly focus on composite transducers and micromachined transducers. In order to facilitate the determination of the operating frequency and reduce undesired energy loss, particular studies on spurious resonance suppression for air-coupled transducers are called for.

In this paper, a suppression approach of spurious resonance in air-coupled transducers is proposed through the adjustment of equivalent parameters by pre-tightening force. The paper is organized as follows. In Section 2, the modeling method of the electrical impedance characteristic of air-coupled transducers with spurious resonance is described. Then in Section 3, the modeling results and the model-based impedance sensitivity analysis to study the mutual interference between adjacent resonances is given in detail. Based on the analysis results, the prestress method is introduced to regulate and suppress the spurious resonance by adjusting the equivalent parameters. Subsequently, experimental results of the beneficial effect of pre-tightening force on the electrical impedance and the vibration response of an air-coupled transducer with spurious resonance are presented. Finally, Section 4 summarizes the important results and the benefits of this work.

## 2. Equivalent Modeling of Air-Coupled Transducers with Spurious Resonance

### 2.1. Equivalent Model

**Figure 1:**
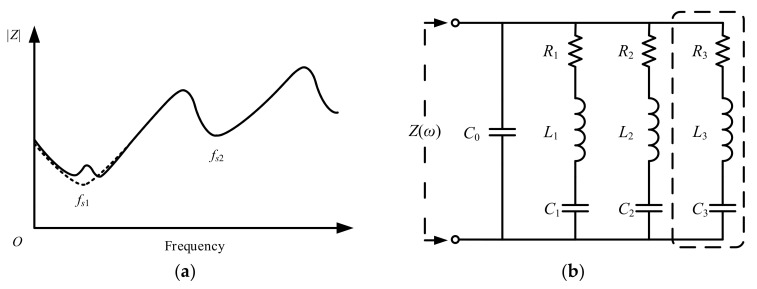
(**a**) Impedance magnitude of air-coupled transducer with double resonances (dotted line) and triple resonances including spurious resonance (solid line); (**b**) Butterworth–Van Dyke (BVD) equivalent circuit with multiple branches (additional branch in the dashed box is required to model the impedance with spurious resonance).

Impedance plots reflect the basic electrical characteristic and can provide valuable information for quality control during the manufacturing process of transducers [9]. The impedance magnitude of an air-coupled transducer with double resonances (dotted line) and that with additional spurious resonance (solid line) are illustrated in Figure 1a. The Butterworth–Van Dyke (BVD) equivalent circuit shown in Figure 1b is mainly used to model the electrical impedance characteristics of transducers. Three is one static branch and *n* series resistance-inductance-capacitance (RLC) branches corresponding to *n* resonances in the equivalent circuit. In each individual branch, *R_n_*, *L_n_*, *C_n_* are equivalent resistance, inductance and capacitance, representing mechanical loss, inertial mass and elastic compliance, respectively [24]. For a transducer with double resonances, a circuit with two series RLC branches can provide satisfactory impedance modeling accuracy. However, for the impedance with the interference of spurious resonance, additional series RLC branch (as dashed box shown in Figure 1b) is required to give a fine representation of the impedance characteristic with spurious resonance.

For the equivalent circuit with three series RLC branches in Figure 1b, the impedance *Z*(*ω*) can be calculated by

(1) $$Z\left(\omega \right)=\frac{1}{Y\left(\omega \right)}=\frac{1}{Y_{C_{0}}\left(\omega \right)+\sum_{n=1}^{3}Y_{n}\left(\omega \right)},$$

where ω is angular frequency, *Y*(*ω*) is the admittance, *Y_C_*_0_(*ω*) is the admittance of static capacitance *C*_0_,

(2) $$Y_{C_{0}}\left(\omega \right)=i\omega C_{0},$$

and *i* is imaginary unit, *Y_n_*(*ω*) is the admittance of the *n*-th series branch,

(3) $$Y_{n}\left(\omega \right)=\frac{1}{R_{n}+i\omega L_{n}+\frac{1}{i\omega C_{n}}},$$

*n* = 1, 2, 3.

In our previous study [15], main focus was given to the impedance of transducers with widely spaced double resonances. On a reasonable assumption of no mutual interference between adjacent resonances, equivalent parameters of the two series RLC branches are calculated independently,

(4) $$R_{n}=\frac{1}{G_{max}},$$

(5) $$L_{n}=\frac{R_{n}}{\omega_{2}-\omega_{1}},$$

(6) $$C_{n}=\frac{1}{\omega_{s}^{2}L_{n}},$$

(7) $$C_{0}=\frac{B_{\omega_{x}}}{\omega_{x}}+\sum_{n=1}^{N}\frac{L_{1}-\frac{1}{\omega_{x}^{2}C_{n}}}{R_{n}^{2}+{\left(\omega_{x}L_{n}-\frac{1}{\omega_{x}C_{n}}\right)}^{2}},$$

where *ω*_1_ and *ω*_2_ are frequencies at which the susceptance arrives at its maximum and minimum value, respectively, matching the frequency points of half maximum conductance (*G*_max_), *ω_x_* is one of the frequency points with a susceptance value *B_ωx_*, *N* is the number of RLC branches.

**Figure 2:**
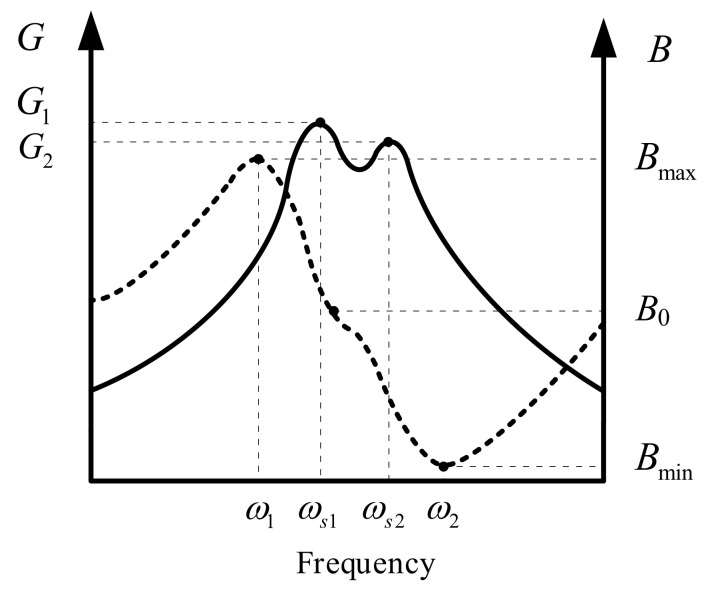
Conductance G (in solid line) and susceptance B (in dotted line) of transducer with closely spaced adjacent resonances.

However, for the impedance, in case spurious resonance exists, it is better to take the coupling effect between closely spaced adjacent resonances into consideration when calculating the initial values of equivalent parameters. As shown in Figure 2, the two adjacent resonances interact with each other, leading to distorted conductance (*G*) and susceptance (*B*). For simplicity, the weaker resonance is supposed to be the spurious resonance. As a result of distortion, the frequency of maximum *G* no longer corresponds to the frequency of zero *B* (*B*_0_) which brings trouble to the direct determination of optimal operating frequency of transducers. In order to facilitate the modeling of transducers with spurious resonance, a method of an initial estimate of the equivalent parameters of the impedance model with spurious resonance is presented below. For the equivalent resistance *R_n_*, *G*_max_ is substituted by local maximum *G*_1_ and *G*_2_ when calculating *R*_1_ and *R*_2_, respectively, based on Equation (4). For the spurious resonance without visible *G*_max_, it is advisable to take the |*Z*|_min_ as a substitution. As for calculating *L_n_* using Equation (5), the upper half-power frequency of the lower resonance and the lower half-power frequency of the upper resonance are invisible due to the interaction between adjacent resonances. As reported in [9], the upper half-power frequency of the lower resonance can be deduced from the 50% bandwidth based on the frequency of *G_max_* and lower half-power frequency. Additionally, the lower half-power frequency of the upper resonance can be similarly estimated. As a workaround, the same pair of half-power frequency points *ω*_1_ and *ω*_2_ can be used to calculate the initial *L_n_*. Afterwards, *C*_1_ and *C*_2_ are derived from Equation (6) by replacing *ω_s_* with local maximum *G* frequencies *ω_s_*_1_ and *ω_s_*_2_, respectively. *C*_0_ can be calculated based on the above results using Equation (7). The rough parameters obtained here will serve as initial values for further parameters optimization to give a better approximation to the measurement impedance.

### 2.2. Equivalent Parameters Estimation

To facilitate the estimation of equivalent parameters, the objective function needs to be formulated elaborately. The impedance magnitude (|*Z*|) and phase (*T*) are taken into consideration in the objective function as a result of their inherent relation with all the equivalent parameters to be estimated. The objective function considering both the sum of relative square errors and correlation coefficients between measured and estimated magnitude and phase of impedance is defined as below,

(8) $$\begin{array}{c} F=\mu \frac{1}{M}\overset{M}{\underset{m=1}{\sum }}\left({\left(\frac{{\left|Z\right|}_{mea}\left(f_{m}\right)-{\left|Z\right|}_{est}\left(f_{m}\right)}{Z_{mea}\left(f_{m}\right)}\right)}^{2}+{\left(\frac{T_{mea}\left(f_{m}\right)-T_{est}\left(f_{m}\right)}{T_{mea}\left(f_{m}\right)+\theta }\right)}^{2}\right)\;\;\;\;\\ \;\;\;\;+\lambda \frac{1}{\left(\left(\mathrm{corrcoef}\left({\left|Z\right|}_{mea},{\left|Z\right|}_{est}\right)+\mathrm{corrcoef}\left(T_{mea},T_{est}\right)\right)+2\right)}, \end{array}$$

where |*Z*|*_est_* and |*Z*|*_mea_* are the estimated and measured impedance magnitude (in logarithmic form), respectively, *T_est_* and *T_mea_* are the estimated and measured phase angle of impedance, respectively, *corrcoef* is a correlation coefficient function, *M* is the total number of measured frequency points over the frequency range of interest, *μ* and *λ* are constant coefficient used to balance the two terms in the fitness function, *θ* is an compensation angle. The value of *θ* is set as 180° to circumvent near-zero denominator when computing the relative error of *T*.

The optimum parameters are often determined with the help of stochastic algorithms such as genetic algorithm (GA), particle swarm optimization, etc. [25]. In this issue, hybrid optimization scheme combined stochastic algorithm (GA) and local optimization solver is utilized to expedite the optimization process. After GA is run for certain number of generations to arrive at the neighborhood of the optimum values, local optimization solver is operated to reach the optimum points quickly. MATLAB optimization toolbox provide appropriate implementation of hybrid optimization. The local optimization solver is chosen as *fmincon* function and default algorithm ‘interior-point’ is employed. GA parameters are set with a maximum generation of 100 and a population number of 200. The parameters search space is constrained within 0~10 times of the initial values. The mutation and crossover rates are set to 0.2 and 0.8, respectively.

## 3. Experiments and Results

### 3.1. Transducer Configuration and Experiment Setup

**Figure 3:**
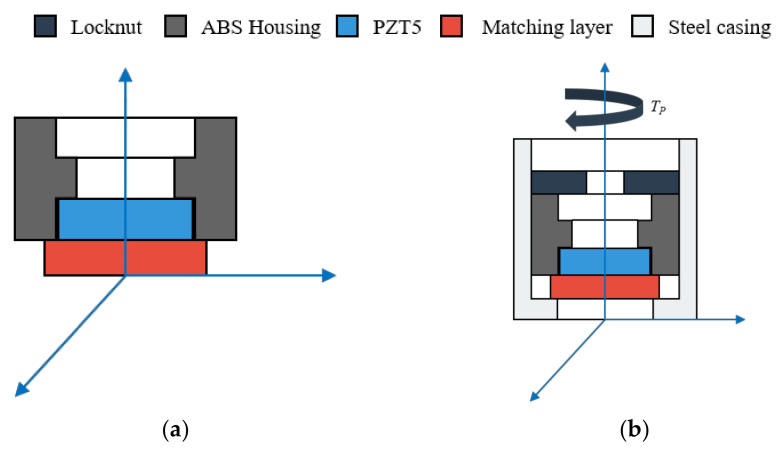
Schematic diagram of (**a**) typical air-coupled transducer; (**b**) prestressed air-coupled transducer.

A homemade air-coupled transducer applied to gas flow measurement shown in Figure 3a is used in this research. A piezoceramic disc serves as the elemental component of air-coupled transducer to convert electrical energy into mechanical vibration and vice versa. The fundamental radial mode of piezoceramic disc is utilized considering both measurement resolution and severe high-frequency attenuation in gas medium. For the purpose of alleviating acoustic impedance mismatch, an acoustic matching layer is inserted between the piezoceramic disc and the medium and is bonded by glue. As a common practice [26], the thickness of the matching layer is tailored to quarter-wavelength around the radial mode fundamental frequency of the piezoceramic disc. Then a transducer is made after the matched piezoceramic is adhered and encapsulated into an ABS housing. The operating frequency of the transducer is around 200 kHz.

Additionally, a prestress scheme is applied to regulate and suppress the spurious resonance in the air-coupled transducer. As shown in Figure 3b, the transducer is fitted to a steel casing with a locknut screwed in the back. A steel casing protects the transducer from environmental corrosion. Besides, the assembling way of the steel casing provides access to the adjustment of the transducer boundary conditions. Additional boundary constraint can be imposed on the peripheral acoustic radiation surface using the locknut. The preload force on the front of the matching layer applied by the pre-tightening torque *T_p_* on the locknut is controlled using a torque wrench (Pake TLB type with a maximum capacity of 5 N∙m and an accuracy within ± 3%). In the following experiments, the potential of spurious resonance control based on prestress method is exploited.

**Figure 4:**
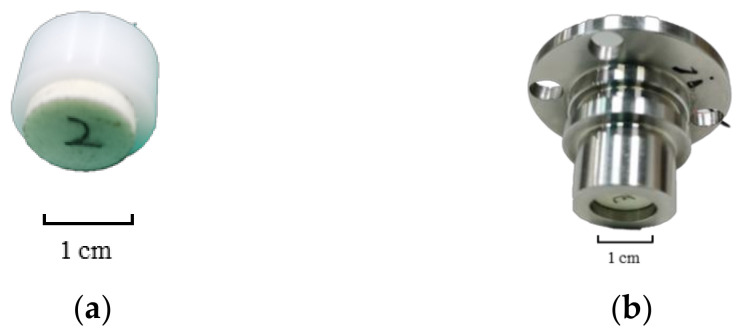
Home-made air-coupled transducer (**a**) without pre-tightening; (**b**) with pre-tightening.

In order to work on the suppression of spurious resonance, the prestress effect was experimentally studied on the electrical impedance and the vibration response of transducers with and without pre-tightening force (as shown in Figure 4). Firstly, the prestress effect on the electrical impedance characteristic was studied. The impedance of transducers was measured with 201 frequency points in the frequency range of interest 150–250 kHz using a digital electrical bridge (TH2826A, Tonghui Electronic Co. Ltd., Changzhou, China). Furthermore, the prestress effect on the vibration response of transducers was also investigated. The vibration velocity of the center on the radiation surface was measured by a laser doppler vibrometer (Polytec NLV-2500, Polytech GmbH, Waldbronn, Germany).

### 3.2. Equivalent Parameters Estimation Results

**Figure 5:**
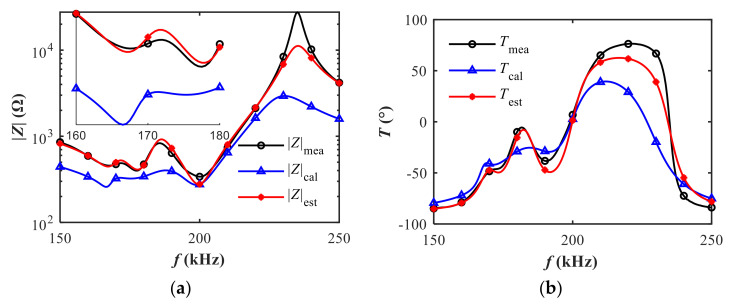
Comparison of model-based impedance based on calculated and estimated parameters: (**a**) magnitude; (**b**) phase.

**Figure 6:**
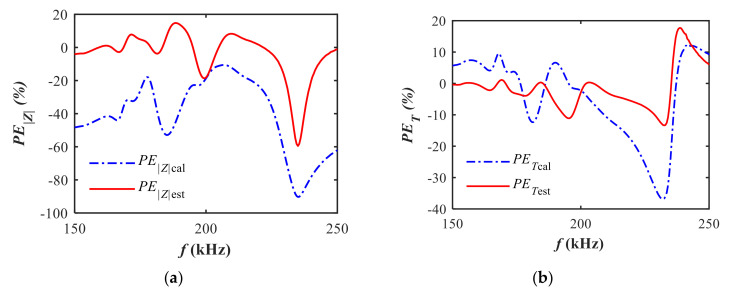
Comparison of percentage errors of impedance based on calculated and estimated parameters relative to the measurements: (**a**) magnitude; (**b**) phase.

The modeling method in Section 2 is used to model the measured electrical impedance of the air-coupled transducer with spurious resonance. Initial values of equivalent parameters are calculated following the method in Section 2.1. Afterwards, refined parameters are further obtained based on the GA optimization method in Section 2.2. For the sake of clarity, the markers of each line in Figure 5 were plotted at an interval of 200 measurement points. As shown in Figure 5, a satisfactory modeling accuracy of the impedance is obtained using the estimated parameters based on the GA method. In contrast, the calculated impedance based on the initial values of equivalent parameters affords a worse representation of the measured impedance. Improved modeling accuracy over the frequency band of interest is achieved as illustrated in Figure 6. The percentage errors (*PE*) between the measured impedance and the model-based impedance based on the initial values and the estimated results are calculated by,

(9) $$PE_{\left|Z\right|}=\frac{{\left|Z\right|}_{mea}\left(m\right)-\left|Z\right|\left(m\right)}{Z_{mea}\left(m\right)},$$

(10) $$PE_{T}=\frac{T_{mea}\left(m\right)-T\left(m\right)}{T_{mea}\left(m\right)+\theta },$$

The overall modeling accuracy of impedance is also examined by the relative root-mean-square error (*rRMSE*),

(11) $$rRMSE\left(\left|Z\right|\right)=\sqrt{\frac{1}{M}\sum_{m=1}^{M}PE_{\left|Z\right|}^{2}\left(m\right)},$$

(12) $$rRMSE\left(T\right)=\sqrt{\frac{1}{M}\sum_{m=1}^{M}PE_{T}^{2}\left(m\right)},$$

Quantitatively, the *rRMSE* of magnitude decreases from 46.0% to 15.1% while the *rRMSE* of phase decreases from 13.9% to 6.9% after the refinement of equivalent parameters based on the GA method. The modeling accuracy in the frequency band of series resonance is better than that in the frequency band of parallel resonance. Specifically, the *rRMSE* of magnitude arrives at 7.6% between 150 and 210 kHz and 21.9% between 210 and 250 kHz. For conductance, the *rRMSE* reaches 11.7% between 150 and 210 kHz and 117.2% between 210 and 250 kHz. As we focus more on the series resonance frequency band where spurious resonance occurs, the proposed method can provide acceptable accuracy on modeling the electrical impedance with spurious resonance.

As shown in Table 1, the estimated values have changed noticeably compared to the initial values. The percentage change of the parameters in *R*_1_*L*_1_*C*_1_ and *R*_2_*L*_2_*C*_2_ branches is larger than that in *R*_3_*L*_3_*C*_3_ branches. This can be ascribed to the low accuracy of the initial estimate of the parameters in *R*_1_*L*_1_*C*_1_ and *R*_2_*L*_2_*C*_2_ branches as a consequence of the strong coupling effect between the two closely spaced resonances.

### 3.3. Mutual Interference between Adjacent Resonances

**Figure 7:**
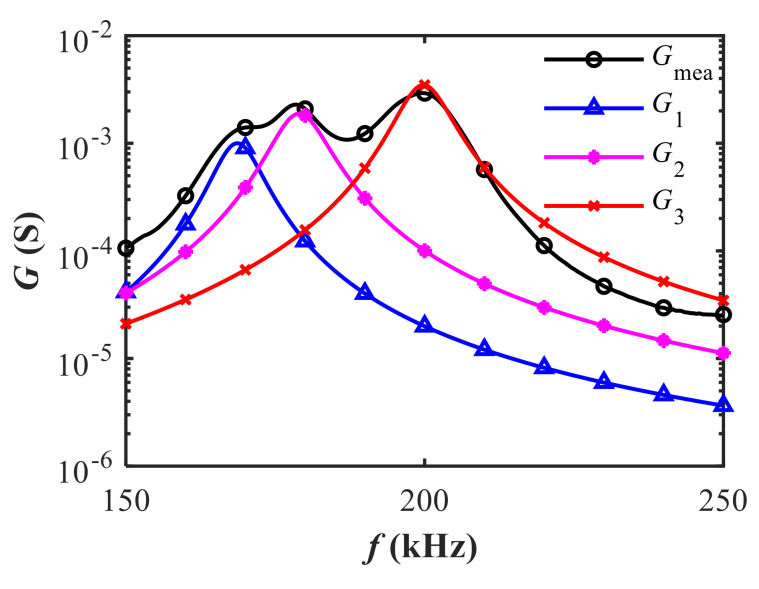
The measured conductance and the calculated conductance of three individual resonances based on the estimated results.

The conductance *G* of each individual resonance is calculated based on the real part of Equation (3). As shown in Figure 7, the first two peaks (*G*_1_ and *G*_2_) interact with each other due to the close frequency spacing between the two resonances. The coupling effect distorts the actual conductance and gives rise to the spurious resonance. The occurrence of the spurious resonance makes the boundary between different resonances so blurred that it brings trouble to the determination of the optimal operating frequency. Besides, the influence of spurious resonance on the performance of air-coupled transducers remains unrevealed.

In order to gain insight into the internal relation between the model-based electrical impedance and the equivalent parameters, the sensitivity analysis is performed using the estimated equivalent parameters obtained in the previous section (in Table 1). Thereby, the effect of equivalent parameter variations on the neighboring electrical impedance is carefully investigated to reveal mutual interference between adjacent resonances.

**Figure 8:**
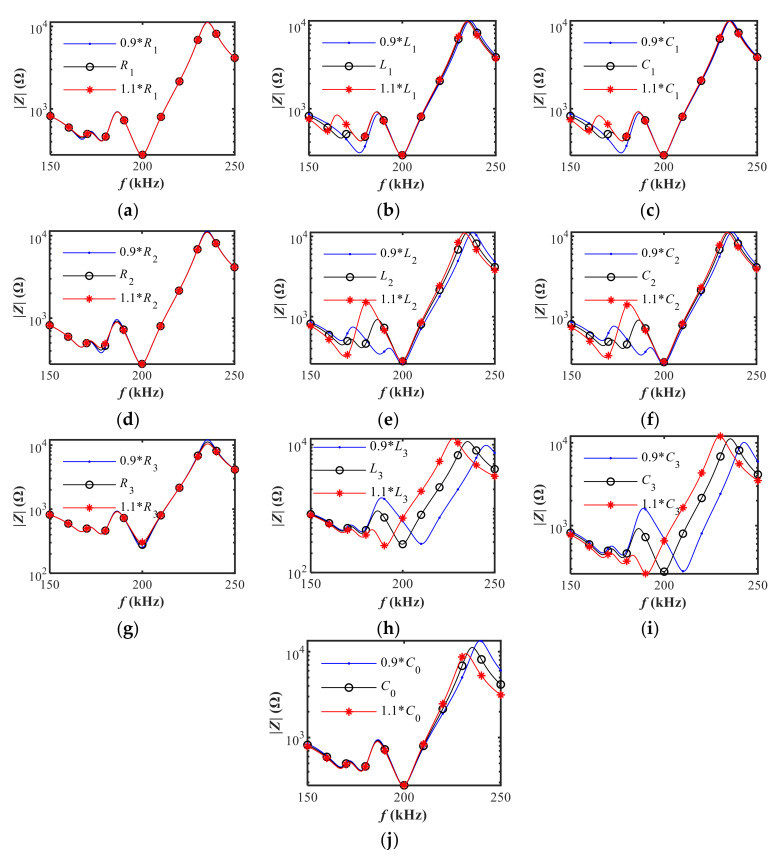
The effect of equivalent parameter variations on the electrical impedance magnitude: (**a**) *R*_1_; (**b**) *L*_1_; (**c**) *C*_1_; (**d**) *R*_2_; (**e**) *L*_2_; (**f**) *C_2_*; (**g**) *R*_3_; (**h**) *L*_3_; (**i**) *C*_3_; (**j**) *C*_0_.

The equivalent parameters were manually varied ±10% on the basis of the original estimated value. The model-based electrical impedance based on Equation (1) is illustrated in Figure 8. On the whole, the impedance magnitude is more susceptible to *L_n_* and *C_n_* than *R_n_*, and the variations of *L_n_* and *C_n_* have similar influence on the impedance. Three resonances are irregularly distributed in the frequency band of interest corresponding to the three series RLC branches in the equivalent circuit. The third resonance is relatively independent from the other two resonances due to the wide frequency spacing. As a result, the variation of *L*_3_ and *C*_3_ barely influences the impedance of the other two resonances. Apparently, the parameter variations exert more influence on the closer resonance. Generally, the coupling effect between adjacent resonances decreases as the frequency interval between the two resonances increases. Mutual interference between adjacent resonances is no longer nonnegligible especially when the two resonances are closely spaced. The first two closely spaced adjacent resonances corresponding to *R*_1_*L*_1_*C*_1_ and *R*_2_*L*_2_*C*_2_ branches are coupled with each other, among which the dominant resonance is main resonance (*R*_2_*L*_2_*C*_2_) while the weaker one is spurious resonance (*R*_1_*L*_1_*C*_1_). The coupling between the adjacent series *R*_1_*L*_1_*C*_1_ and *R*_2_*L*_2_*C*_2_ branches can be altered by a slight variation of *L*_1_, *C*_1_, *L*_2_, and *C*_2_. It is worth noting that the spurious resonance can be weakened or even be eliminated under particular parameters combination.

With the help of sensitivity analysis, it was found feasible to regulate spurious resonance by tuning the equivalent parameters. However, the equivalent parameters are difficult to adjust separately in practice as the sensitivity analysis was done for individual parameter. Herein a prestressed method is proposed to simultaneously adjust the equivalent parameters through changing the boundary conditions of transducers and hence regulates and suppresses the spurious resonance.

### 3.4. Prestress Effects on the Electrical Impedance and the Vibration Response

**Figure 9:**
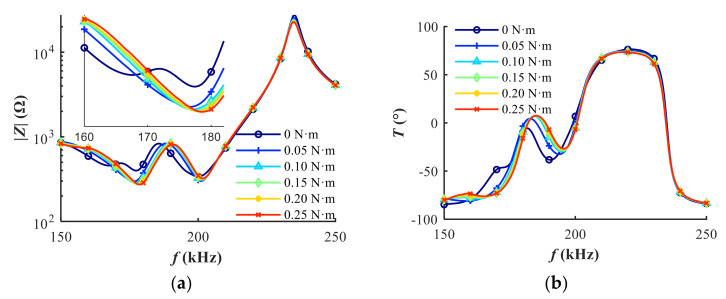
The effect of pre-tightening force on the electrical impedance characteristic: (**a**) magnitude; (**b**) phase.

**Figure 10:**
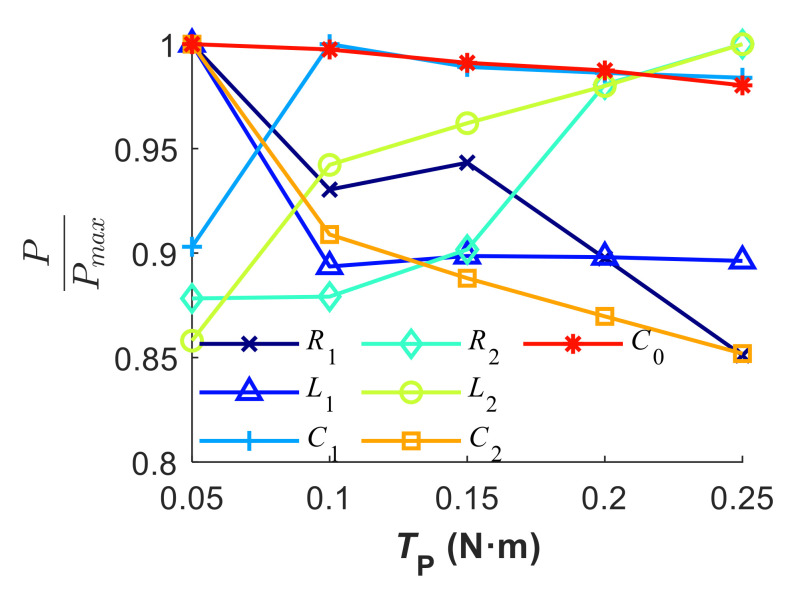
Drift of the equivalent parameters under different loads.

The parametric analysis permits the regulation of spurious resonance through the variation of equivalent parameters. Accordingly, a pre-tightening approach is applied to change the equivalent parameters. Additionally, the effect of pre-tightening force on the electrical impedance characteristic is studied experimentally. As shown in Figure 9, the spurious resonance situated around 170 kHz disappears as soon as the pre-tightening force is initially loaded. As the torque increases continuously, the resonance frequency increases. Meanwhile, the magnitude of impedance around resonance frequencies reduces. The frequency shift and impedance variation around the first two resonances is larger than that of the third resonance. It is worth noting that the exact amount of pre-tightening force on the piezo and the matching layer is actually difficult to estimate when the torque is applied by the torque wrench. The friction coefficient between the lock nut and the steel casing depends on the materials and lubrication. Additionally, the ABS housing contacts the piezo and the matching layer. However, it can be safely concluded that the pre-tightening force is positively related with the applied torque based on the fact that the frequency drift is monotonic as the applied torque increases. As shown in Figure 10, the estimated equivalent parameters change about 15% when the pre-tightening force increases from 0.05 to 0.25 N∙m. The results show that the pre-tightening force on the regulation of spurious resonance in the electrical impedance is efficient.

**Figure 11:**
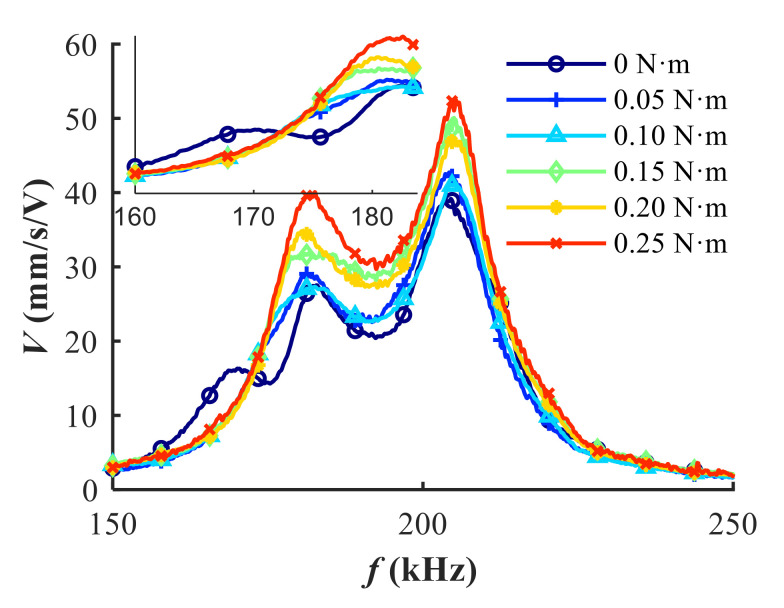
The effect of the pre-tightening force on the vibration velocity of the center on the radiation surface of the transducer.

In addition to the electrical impedance, special attention is given to the transmitting response performance of transducers under different pre-tightening forces. In order to figure out the influence of pre-tightening force on the transducer’s performance, the vibration measurement experiments based on laser doppler principle were conducted. The vibration velocity of the center on the radiation surface of the transducer was measured with 2561 FFT (fast Fourier transform) lines in the frequency range of interest (150–250 kHz) under the excitation of ±10 V white noise. For the sake of clarity, the markers of each line in Figure 11 were plotted at an interval of 200 measurement points. As shown in Figure 11, the spurious resonance located around 170 kHz vanishes immediately when the pre-tightening force is applied. The vibration velocity increases remarkably as the pre-tightening force increases. The two major resonances are witnessed approximating 45.6% and 33.9% increasements of vibration velocity after the suppression of spurious resonance under the applied torque 0.25 N∙m. Therefore, the use of pre-tightening force is beneficial to the transmitting response performance of transducers. On the other hand, the existence of spurious resonance indicates distorted electrical impedance characteristic and deteriorated performance in the transmitting response. It is worth noting that the magnitude of pre-tightening force should not exceed the compression strength of the weakest part of the transducer (typically the matching layer).

As for the generation mechanism of spurious resonance, it can be ascribed to the nonuniform distribution of the stress on the sensitive elements i.e., the piezoceramic disc and the matching layer. The stress may come from the bonding interface between the piezo disc and the matching layer or the housing. The energy transfer between the piezoceramic disc and the matching layer is enhanced when the pre-load is applied. Since the pre-tightening force is applied only on the outer edge of the matching layer and the piezoceramic disc, the vibration of the central area on the radiation surface is kept free and suffers negligible constraint. In consequence, improved performance is achieved after the spurious resonance disappears under pre-tightening force.

## 4. Conclusions

In this study, the electrical impedance characteristic of air-coupled transducer with spurious resonance is modeled based on the BVD equivalent circuit. Subsequently, mutual interference between adjacent resonances is identified explicitly using model-based sensitivity analysis. The analysis reveals that the coupling effect decreases rapidly as the frequency spacing between two adjacent resonances increases. Moreover, it was found feasible to regulate spurious resonance by the adjustment of equivalent parameters. Thereafter, a prestressed method is proposed to tune the equivalent parameters simultaneously through changing the boundary conditions of transducers and hence regulates and suppresses the spurious resonance. Experiments were conducted to study the variations of the electrical impedance and the vibration velocity in response to the pre-tightening force. The results demonstrate that the spurious resonance in the electrical impedance is pretty sensitive to the pre-tightening force. As soon as the pre-tightening force is applied, the spurious resonance vanishes. Approximate 45.6% and 33.9% increases of vibration velocity were observed after the suppression of spurious resonance under the applied torque 0.25 N∙m. The measurement results of the vibration velocity under different pre-tightening forces verify the effectiveness of the proposed method to regulate and suppress the spurious resonance and thus improves the performance of the air-coupled transducer.

## Figures and Tables

**Table 1:** Calculated and estimated equivalent parameters.

	Calculated Values	Estimated Values by GA	Percentage Change (%)
*R*_1_	825.9 Ω	997.9 Ω	20.82
*L*_1_	26.29 mH	19.28 mH	−26.65
*C*_1_	34.34 pF	46.18 pF	34.48
*R*_2_	436.9 Ω	529.6 Ω	21.23
*L*_2_	2.575 mH	8.948 mH	247.5
*C*_2_	308.7 pF	88.34 pF	−71.39
*R*_3_	341.4 Ω	287.0 Ω	−15.92
*L*_3_	4.347 mH	5.046 mH	16.08
*C*_3_	146.4 pF	125.7 pF	−14.11
*C*_0_	972.9 pF	501.1 pF	−48.50
